# Development of metal-free layered semiconductors for 2D organic field-effect transistors

**DOI:** 10.1039/d1cs00497b

**Published:** 2021-08-26

**Authors:** David Burmeister, Matthias G. Trunk, Michael J. Bojdys

**Affiliations:** Institut für Chemie, Humboldt-Universität zu Berlin Brook-Taylor-Str. 2 12489 Berlin Germany m.j.bojdys.02@cantab.net; Integrative Research Institute for the Sciences Adlershof, Humboldt-Universität zu Berlin Zum Großen Windkanal 2 12489 Berlin Germany; Department of Chemistry, King's College London, Britannia House Guy's Campus 7 Trinity Street London SE1 1DB UK

## Abstract

To this day, the active components of integrated circuits consist mostly of (semi-)metals. Concerns for raw material supply and pricing aside, the overreliance on (semi-)metals in electronics limits our abilities (i) to tune the properties and composition of the active components, (ii) to freely process their physical dimensions, and (iii) to expand their deployment to applications that require optical transparency, mechanical flexibility, and permeability. 2D organic semiconductors match these criteria more closely. In this review, we discuss a number of 2D organic materials that can facilitate charge transport across and in-between their π-conjugated layers as well as the challenges that arise from modulation and processing of organic polymer semiconductors in electronic devices such as organic field-effect transistors.

## Abstract 2

Metal-free 2D covalent organic materials transport charges along and in-between π-conjugated layers. Here, we look at the prospects of graphitic carbon nitrides and covalent organic frameworks as 2D semiconductors “beyond graphene and silicon”.

## Graphical abstract

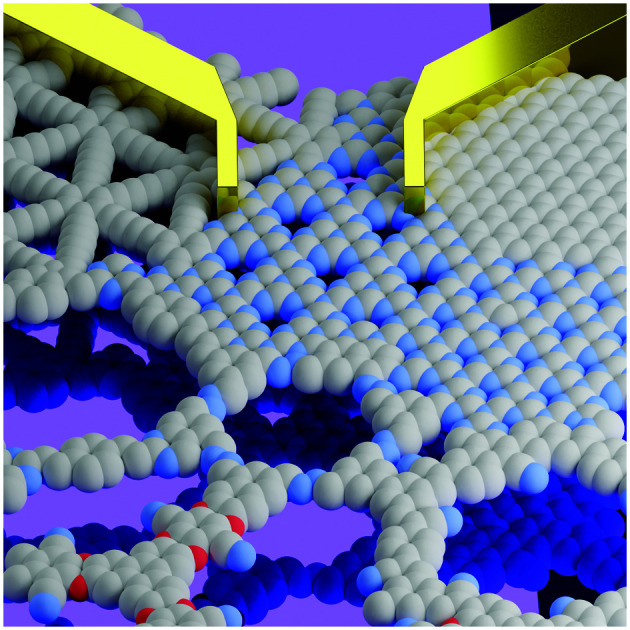

## 1. From 0 to 1 to 2D

Silicon transistors are omnipresent in our lives, and they can be thought of as electrically controlled on/off switches. Their integration into circuits and successively smaller-sized logic gates has given rise to an exponential growth of computing power over the last 50 years. While silicon is an abundant material and its processing is one of the most sophisticated technologies ever developed, these processes are also energy-intensive, accounting for 2% of annual consumed energy in the US,^1^ and up to 30% of microchip fabrication costs.^2^

Commercial silicon technology can currently mass-produce feature sizes of 5 nm, but short-channel effects,^3^ no increase in clock frequency, and extremely costly lithography become increasingly deterrent.^4^ According to the International Technology Roadmap for Semiconductors (ITRS), these circumstances render further miniaturization of silicon technologies economically unfeasible.^5^ Additionally, critical raw materials are used in the doping of silicon. The goal is to find new, high-performing semiconductors that are able to adopt the role of silicon at these small scales and are not reliant on critical raw materials. From a materials design perspective, the chemical bias set-up in dense crystalline silicon phases is usually long-lived and stable, but leaves very little scope for chemical modifications of the bulk.^6^ Furthermore, silicon lacks flexibility and its high weight, opacity, and low compatibility with biological tissues render it unsuitable for various emerging areas of application such as wearable, breathable electronics.

To find suitable materials that match these criteria, organic molecular (0D) and linear polymeric semiconductors (1D) are widely researched.^7,8^ However, these materials often suffer from low structural order, low amounts of charge carriers and high concentrations of defect sites, leading to low mobility and high injection barriers. Some of these shortcomings were resolved by spatially defined doping and attempts at increasing order in these systems by point-anchoring, supramolecular assembly or by liquid crystallinity. However, the inherent free movement of organic molecules and polymer chains in these systems tends to break down the desired chemical bias introduced by dopants and, over time, leads to reduced lifetimes and efficiencies of organic electronic devices.^9^ Additionally, ambient conditions can have deteriorating effects on the chemical structures and charge transport properties of the involved organic materials.^10,11^ The resulting low performance and possible short-channel effects make miniaturization of devices based on conventional organic (*i.e.* molecular and polymeric) semiconductors challenging.^3,12^

Overcoming the limitations inherent to silicon and conventional organic semiconductors is the prime incentive for the development of two-dimensional (2D) covalent organic semiconducting materials.^13^ To reach this ambitious goal, materials need to exhibit (i) a band gap (0.3–3 eV), (ii) structural as well as energetic order (crystallinity), (iii) ambient stability, (iv) 2D morphology and (v) high charge carrier mobility. Material classes with the potential to satisfy these requirements are covalent organic frameworks (COFs) and carbon nitrides. The majority of COFs are crystalline, fully conjugated layered materials synthesized from one or several organic building blocks.^14^ This modular makeup allows atomically precise structural engineering on two levels in order to tune the electronic properties of the resulting material.

**Fig. 1:**
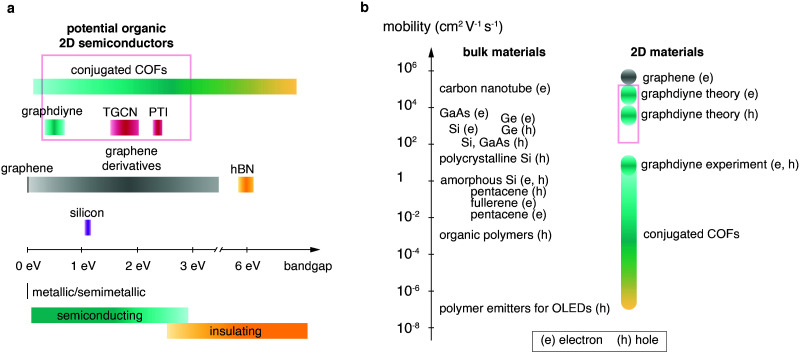
Overview over semiconducting materials plotted in relation to (a) band gap and (b) mobility (e, electrons; h, holes). Candidates for organic 2D semiconductors are highlighted by red boxes. Fig. 1b was adapted from Tiwari and Greenham^164^ with permission from Springer Nature.

Firstly, the monomer(s) can be fashioned with specific functional groups. Secondly, the choice of linking chemistry determines the nature of communication between the building blocks of these frameworks. For example, in-plane π-conjugation and layering of aromatic domains determines emerging functionalities such as (semi-)conductivity, optical properties and catalytic activity. Recent approaches based on carbon–carbon couplings have produced highly conjugated structures including materials such as graphdiyne, for which monolayers exhibiting charge carrier mobilities on the order of 10.000 cm^2^ V^−1^ s^−1^ and a direct bandgap of 0.46 eV are predicted (see Table 1 at the end of this document).^15^ The sizable bandgap of >0.3 eV and high charge carrier mobilities make graphdiyne a promising candidate for high-performance organic 2D transistors.^16^ Carbon nitride materials, such as triazine-based graphitic carbon nitride (TGCN), with a high N/C ratio, are expected to exhibit high electron affinity enabling their application as chemically and thermally stable, ambipolar semiconductors.^17^ The bandgap of TGCN has been estimated to be <1.6 eV, making it an additional candidate for an organic 2D semiconductor.^18^ Both layered COFs and layered carbon nitrides can be potentially exfoliated to covalently bonded layers of atomic thickness, *i.e.* monolayers. Potential organic 2D semiconductors are shown in Fig. 1 and compared to their current competitors. Covalent organic materials are arguably less affected by dopant drift and migration. Depending on pore size and stacking distances the migration of large atomic dopants such as iodine can be sufficiently hindered. This could enable doping at specific sites such as the electrode–semiconductor interface in order to enhance charge injection.^19,20^

**Table 1:** Experimental and theoretical electrical mobility values of covalent organic materials and graphene

Material	Material class	Vertical dimension (method)	Mobility (charge carrier, method)	Ref.
Graphene	2D semimetal	0.4 ± 0.3 nm (peak force tapping AFM)	200 000 cm^2^ V^−1^ s^−1^ (electron, suspended, 5 K)	160–162
Graphdiyne	2D semiconductor		10 000 cm^2^ V^−1^ s^−1^ (electron, theory)	15
Porphyrin-imine COF	Bulk semiconductor	—	8.1 cm^2^ V^−1^ s^−1^ (hole, TOF)	163
Benzodithiophene-imine COF	Bulk semiconductor	1.8 nm (AFM)	3 × 10^−6^ cm^2^ V^−1^ s^−1^ (hole, FET)	113
Porphyrin-imine COF on hBN (COF-366@hBN)	Bulk semiconductor	2.34–4.56 nm (AFM)	0.015 × 10^−6^ cm^2^ V^−1^ s^−1^ (hole, FET)	116
Porphyrin-dihydroxyphenylene COF	Bulk semiconductor	0.7 nm (AFM)	1.3 × 10^−6^ cm^2^ V^−1^ s^−1^; 1.6 × 10^−4^ cm^2^ V^−1^ s^−1^ (I_2_-doped)	147
Benzodithiophene-boronate ester COF	Bulk semiconductor	80 nm (cross-section SEM)	3 × 10^−7^ cm^2^ V^−1^ s^−1^ (hole-only device)	144
Graphdiyne	Bulk semiconductor	2.9 nm	6.25 cm^2^ V^−1^ s^−1^ (FET)	94
Poly-1,3,5-benzene Suzuki polymer	Bulk semiconductor	—	3.2 cm^2^ V^−1^ s^−1^ (FET)	99
Triazatruxene Suzuki polymer	Bulk semiconductor	2.5–46 nm (AFM)	1.37 cm^2^ V^−1^ s^−1^ (hole, FET)	100

Despite the rapid progress in the field of layered organic semiconductors, the anticipated high-performance organic transistor has not been achieved yet. The devices constructed from layered organic materials exhibit mobilities only scarcely exceeding that of amorphous silicon thin film transistors (0.5–1 cm^2^ V^−1^ s^−1^).^21^ Exploring the present state of the different material classes we notice common challenges, *i.e.* (i) structural and energetic disorder (low crystallinity), (ii) charge carrier anisotropy (intraplane *vs.* interplane charge transport) and (iii) processing of the solids into suitable thin films. In this review we map out the landscape of organic layered semiconductors, the strong points of the individual material types and the challenges the field has to overcome to produce materials applicable in flexible, high-performance, low-cost, low-power electronics.

## 2. Candidates for 2D organic semiconductors

The “graphene family” as defined by Geim *et al.* in 2013 consists of five members, namely graphene, hexagonal boronitride (hBN), borocarbonitride (BCN), fluorographene, and graphene oxide.^22^ To carve out the necessary steps towards efficient organic 2D semiconductors it is important to understand the advantages as well as the shortcomings of these materials.

Graphene is an inorganic semimetal, hence graphene will only be investigated as a new type of electrode with a variable work function (see Section 3.1). Several derivatives of graphene are insulators, *i.e.* graphane (C_1_H_1_), fluorographene (C_1_F_1_), and fluorographane (C_1_H_0.5_F_0.5_).^23^ While still intriguing as possible gate insulators or tunnel barriers they cannot be employed as 2D semiconductors.^24–27^

A commonly employed strategy in the semiconductor industry is to alter the electronic properties of homonuclear lattices. In silicon, for example, heteroatoms are incorporated into the bulk lattice, which is typically referred to as “impurity doping”.^28–31^ Impurity doping of graphene was initially conducted in order to obtain a 2D semiconductor. However, the doping concentration needs to be high and homogeneous enough over the dimensions of the device that no pristine channels of the initial semimetallic material remain. In addition, the introduction of new, polarising elements also introduces new scattering sites for charge carriers. This results in a trade-off between energetic control and deterioration of the extraordinary transport properties of graphene.^32^ Doping of graphene with boron and nitrogen defect sites alters the electronic structure, but the surrounding semimetallic domains typically dominate the electronic properties of the B/N doped graphene in device architectures. Hence, the problem of the absence of an off-state – and therefore poor performance of field-effect transistors based on graphene – remains.^33^ Tailoring the properties of graphene's electronic structure by nanostructuring increased the performance (on/off ratio up to 10^4^) and provided new insights into magnetotransport in graphene, but has not yet reached values to rival existing silicon technology.^34,35^

Graphene oxide has the advantage of being solution-processable, and it is typically viewed as a candidate for metal-free, transparent electrodes. However, the oxidized sites make graphene oxide non-conductive. To regain conductivity, graphene oxide has to be reduced, but reduced graphene oxide films do not yet reach the performance of chemical vapor deposition (CVD) graphene films produced by roll-to-roll processes, or the performance of conventional heavy-metal-based indium tin oxide (ITO) transparent electrodes.^36^

It is evident that the introduction of (few-)site dopants into graphene as a strategy to widen its band gap is highly limited and impractical. An alternative strategy is to synthesize heteroatom-containing structures analogous to graphene in a bottom-up approach.

Hexagonal boron nitride (hBN) – while structurally similar to graphene – is a wide-bandgap semiconductor. In fact, it is one of the most widely employed two-dimensional insulators.^37^ It is anticipated that the electronic properties of ternary layered boron carbon nitrides (BCN) can be tuned from insulating to semimetallic with varying composition.^38^ The partaking elements B, C, N can form homophilic (C–C) as well as heterophilic (C–N, B–N, C–B) bonds, giving rise to many different polymorphs. Quantum chemical calculations show that semiconducting hexagonal BC_*x*_N alloys could form under non-equilibrium conditions, and that annealing can have a major impact on the electronic structure.^38^ An in-depth study of the formation of BCN on a ruthenium catalyst conducted by Lu *et al.* showed the multitude of possible structures and pointed out that the formation of purely two-dimensional BCN alloys is still not well-understood.^39^ Many different post-synthetic modifications of BCN materials resulted in various polymorphs and nanostructures. 2D BCN crystallites, however, have yet to be synthesized in a bottom-up approach.^40,41^

Some graphitic semiconducting materials can be found among binary boron carbide (BC_*x*_) polymorphs. Bulk BC_3_ was reported to be a metallic material with turbostratically disordered layers, and monolayers were predicted to exhibit an indirect band gap.^42–44^ Quantum chemical calculations of boron carbide materials with increasing boron content show that most of these materials exhibit metallic behaviour.^45,46^ An experimental study of boron-rich carbon films synthesized by a hot-press method found various polymorphs with local structural disorder.^47^ A graphitic, layered boron carbide with a direct bandgap has not been synthesized to date.^48^

Overall, the graphene family consists mostly of semimetal or insulating members and does not yet have a promising candidate for an organic 2D semiconductor.

### 2.1 Two-dimensional C_3_N_4_ materials

One field promising the development of new direct-bandgap 2D semiconductors with high environmental stability and intriguing chemically active sites is the field of carbon nitrides.^18,49^

A new synthetic approach entailed the use of solvothermal conditions often applied in inorganic synthesis.^50^ Different solvents such as benzene and hydrazine were applied but the products typically displayed residual –NH–/–NH_2_ groups as can be deduced from IR bands at 3200–3300 cm^−1^.^51,52^ For complete condensation the absence of reducing agents and elevated temperatures were necessary. Experiments in laser-heated diamond-anvil-cells yielded a crystalline C_2_N_2_(NH) phase,^53^ and a stable, chiral carbon nitride polymorph with space group *P*4_3_2_1_2 and mixed sp^2^/sp^3^ bonding.^54^ A synthesis at high temperature under ionothermal conditions using a eutectic salt melt of lithium chloride and potassium chloride for the polycondensation of dicyandiamide was also investigated. The structural analysis of the crystalline product led to the conclusion that the observed product is heptazine-based graphitic carbon nitride (HGCN), but the product was later identified as polytriazine imide (PTI) with intercalated lithium and chloride ions.^55,56^ This product constitutes the first truly graphitic carbon nitride for which the structure was fully resolved. Hence, we want to direct our attention to the synthesis, structure, morphology, and recent results regarding this material in more detail.

#### 2.1.1 Polytriazine imide PTI-MX – the first truly graphitic carbon nitride

The in-plane structure of polytriazine imide consists of triazine cores bridged by nitrogen atoms, but the structure is not fully condensed (Fig. 3a). Within each layer, one third of the triazine cores are substituted by metal ions (M^+^), which are balanced by halide ions (X^−^) intercalated between the layers, hence the nomenclature of PTI-MX. Postsynthetic exchange of the halide and metal ions was investigated to tune the gallery height.^57,58^

Kessler *et al.*^59^ published an extensive study on the formation processes of PTI from different precursors and eutectics, suggesting that the first step towards forming the PTI scaffold is always the formation of melem, *i.e.* triaminoheptazine. Depending on the reaction temperature and whether a closed vessel is used or not, melem can either condense under formation of nitrogen-bridged, one-dimensional heptazine chains, *i.e.* melon, or undergo a ring-opening reaction leading to the assembly of nitrogen-bridged, one-dimensional triazine strands. Further condensation of these strands leads to the formation of PTI. This mechanistic insight will be a good foundation for rationally designing new ionothermal synthesis approaches.

A recent study showed that PTI-LiCl produced under ambient conditions has an orthorhombic structure with a *Cmc*2_1_ space group,^60^ which is a superstructure to the earlier reported hexagonal structure *P*6_3_*cm*.^56^ The authors also demonstrate that higher accessibility of the (001) plane increases the photocatalytic activity, indicating that the active sites are not at defect sites or grain boundaries as observed for inorganic materials, but that the planar structures themselves are catalytically active.^60^

The product morphology is characterised as disordered platelets or hollow tubes consisting of hexagonal prisms with single crystallites on the order of 50 nm.^56,57^ The layers are stacked in an AA′ manner with ion channels running orthogonally to the (001) plane of the crystal structure. Due to the high structural order, for the first time it was possible to resolve the triazine breathing modes in a Raman spectrum for a condensed truly graphitic carbon nitride material at 1000 cm^−1^ and 680 cm^−1^, using a 325 nm laser.^61^ This enables to control the quality of microscopic amounts of the material on substrates, which is important for device fabrication. Furthermore, it opened the door to follow-up experiments investigating the vibrations of the material in dependence of temperature or dopants. TEM images of monolayers were first recorded by Villalobos *et al.*^62^ This study indicates that PTI indeed is stable as a 2D sheet, opening a door towards experiments with the 2D crystal of PTI. Another complementary approach applying NMR and electron diffraction pinned down the positions of hydrogen atoms and chloride ions.^63^ The bandgap of PTI-LiCl was determined to be 2.2 eV, which is 0.5 eV smaller compared to the bandgap of melon, indicating a higher degree of conjugation for PTI-LiCl.^64^

While the existence of PTI proves the possibility of synthesising crystalline, graphitic carbon nitride structures, the presence of –NH– groups and ions inside the structure are unfavourable to achieve a high-mobility organic semiconductor with high energetic order. Hence, the complete condensation to a binary CN material still had to be realised.

#### 2.1.2 The first report of a fully condensed C_3_N_4_ material – triazine-based graphitic carbon nitride

Siller *et al.* reported the synthesis and characterisation of triazine-based graphitic carbon nitride (TGCN), which remains the only crystalline binary C_3_N_4_ system to this day.^18,65,66^ TGCN comprises two-dimensional networks of triazine units bridged by nitrogen atoms. Infrared spectroscopy and elemental analysis showed that – in contrast to PTI-LiCl – only few –NH groups and salt intercalations remained. Overall, the synthesis conditions are extremely similar to the synthesis of PTI-MX.^55^ Dicyandiamide is ground with a eutectic salt mixture and heated to 600 °C in a closed quartz ampule. Further, the high temperature and the presence of decomposition products enable reversible reactions since condensation products such as ammonia remain within the closed system.^67^ The differences to the synthesis of PTI-LiBr are the heating program, which for TGCN entails two successive heating steps, and the reaction time, which is increased to three days. These changes were sufficient to observe a new carbon nitride phase evolving at the walls of the quartz ampules as well as at the gas–molten salt interface.

The macroscopic appearance was described as “shiny flakes” with colours ranging from transparent red for short reaction times to “shiny flakes that are optically opaque” for long reaction times. The structural assignment was conducted by a combination of high-resolution transmission electron microscopy (HRTEM) and powder X-ray diffraction (PXRD) measurements. The observed HRTEM images with hexagonal 2.6 nm periodicity were matched to HRTEM simulations of ABC-stacked TGCN. The synchrotron PXRD measurements further reinforced the PXRD results in showing the (001) reflection (*a* = 0.504 nm). The best fit for the PXRD data was found to be an AB stacking mode. The conflicting observations of TEM and PXRD were interpreted as weak interplane forces enabling different stacking orders. Hence, locally obtained TEM diffraction data does not necessarily reflect the overall observed PXRD data. Increasing the crystallinity of the material is a future goal since large crystallites and their isolation from amorphous phases has not been reported yet. On the basis of solid-state ultraviolet-visible (UV-Vis) spectra the authors reasoned that the optical gap of the structure is less than 1.6 eV. However, it should be noted that the presented density-functional theory (DFT) calculation of single layer TGCN indicates a bandgap of 2.5 eV, and that DFT typically underestimates the band gap.^68^ The discrepancy between DFT and experiment is also reflected by the predicted corrugation of the structure. Geometry-optimized structures by DFT typically show that the triazine cores are not perfectly in-plane (corrugated structure), while structural refinement from diffraction experiments implies a perfectly planar orientation.^18^

In a successive study of the transport characteristics in the as-received films, Noda *et al.* observed that conductivity out-of-plane was higher than in-plane by a factor of 65. The authors concluded that the nitrogen atoms connecting the triazine rings are not fully sp^2^-hybridized, which results in a low degree of conjugation for the as-received material. Thus, transport of charge carriers by interplanar hopping is favoured over intraplanar transport.^69^ Learning about anisotropic properties is crucial for effective device implementation of newly discovered layered materials.

In an attempt to reproduce the synthesis by Siller *et al.*, Suter *et al.* obtained flakes which did not exhibit the same PXRD reflections.^61^ Elemental analysis showed additional amounts of carbon compared to the ideal C_3_N_4_ composition and the Raman spectrum did not exhibit the characteristic triazine breathing modes. Nevertheless, similar HRTEM images as reported by Siller *et al.* were obtained. Bulk chemical analyses showed that the obtained films contained 2.3–2.5 wt% hydrogen. A neutral hydrogen atom attached to the triazine ring of TGCN was proposed to explain the missing triazine breathing mode in the Raman spectrum, missing NH stretches in the FTIR spectrum, the residual hydrogen in the elemental analysis, and the EPR response. The authors further note that the presence of the neutral hydrogen atoms in the structure could induce conducting domains or localized unpaired spins. The discrepancy to the work conducted by Siller *et al.* is addressed, reasoning that slight differences for example in the geometry of the used ovens can be the reason for different obtained product phases. This observation is important since reproducibility of the synthesis is not only determined by the reaction mixture but also by the heating program and the local temperatures in the oven influenced by the location of the heating elements and temperature sensors.

Truly graphitic carbon nitride materials like PTI-MX and TGCN are first promising steps towards the successful elucidation of structure–property-relationships in 2D carbon nitrides. Next to the previously discussed CN phases the realisation of new CN polymorphs predicted by theory is also a subject of further research.^70^

**Fig. 2:**
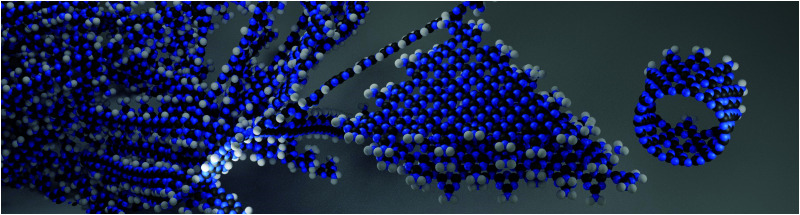
Artistic depiction of the coevolution of different CN phases in the pyrolytic condensation reaction. From the past into the present towards the future – polymeric CNH as discovered 200 years ago (background), condensed TGCN sheets, discovered in 2014 (middle), and tubular structure predicted in 2015 (right).^70^

For the implementation of TGCN into electronic devices, further research must be directed towards increasing the crystalline domain size and isolating the phase-pure material. Current synthetic protocols offer little control of the mixture of co-evolving phases, such as 1D strands, 2D sheets, and predicted tubular structures (Fig. 2, artist's rendition). We adopted the nomenclature recommended by Miller *et al.* for condensed carbon nitride materials and want to recommend it to our quickly growing community, even though we want to stress that in this nomenclature the “g” in “gCN” does not equate to a graphitic material, but to a layered one.^71^ Differentiating “graphitic” from “turbostratic” materials has real-world implications. For example, the difference in the electronic system between a defined graphitic stacking motif with strong interlayer coupling and turbostratic (rotationally faulted) structures that are electronically decoupled can be important as observed in multilayer epitaxial graphene.^72^ We emphasize the importance of a commonly used language with clearly defined terms in a multidisciplinary research field.

### 2.2 Metal-free layered semiconductors from designer molecules

Condensed carbon nitride syntheses start from simple precursors such as dicyandiamide. The repeating units are generated *in situ* and then linked *via* a complex series of condensations requiring high temperatures (500–600 °C). Covalent organic frameworks (COFs) on the other hand are generated from *ex situ* synthesized organic monomers. In most cases, the linking of monomers to frameworks entails the elimination of water and the chemical equilibrium of these condensations is reached at much lower temperatures than in the case of pyrolytically generated carbon nitrides. Typically, bulk COF polymerizations can be carried out at 120–150 °C while the synthesis of COF thin films can be performed at or near ambient temperature. A fundamental advantage of COFs over carbon nitrides is the possibility to design monomers with highly specific functions, which translate into the target frameworks. Additional functionality can arise from the electronic interplay between monomers enabled by conjugated linkages as well as from the linkage motif itself.

#### 2.2.1 Covalent organic framework linkages

Various linkages offer π-conjugation and are associated with different combinations of traits, such as reversibility and chemical stability (Fig. 4). These properties can be exploited in different ways to obtain thin films suitable for device fabrication.

**Fig. 3:**
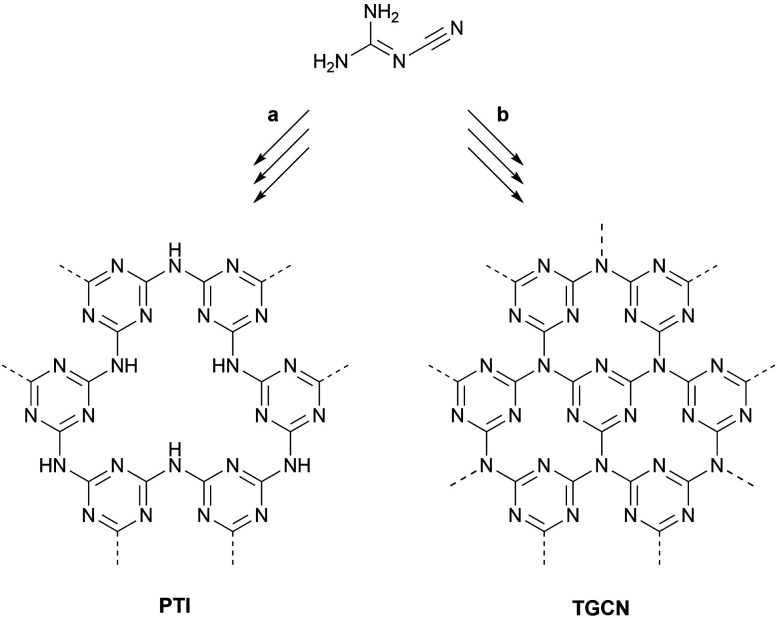
Ionothermal syntheses of PTI and TGCN from dicyandiamide. (a) 400 °C, 12 h; then 600 °C, 48 h. (b) 400 °C, 4 h; then 600 °C, 60 h.

**Fig. 4:**
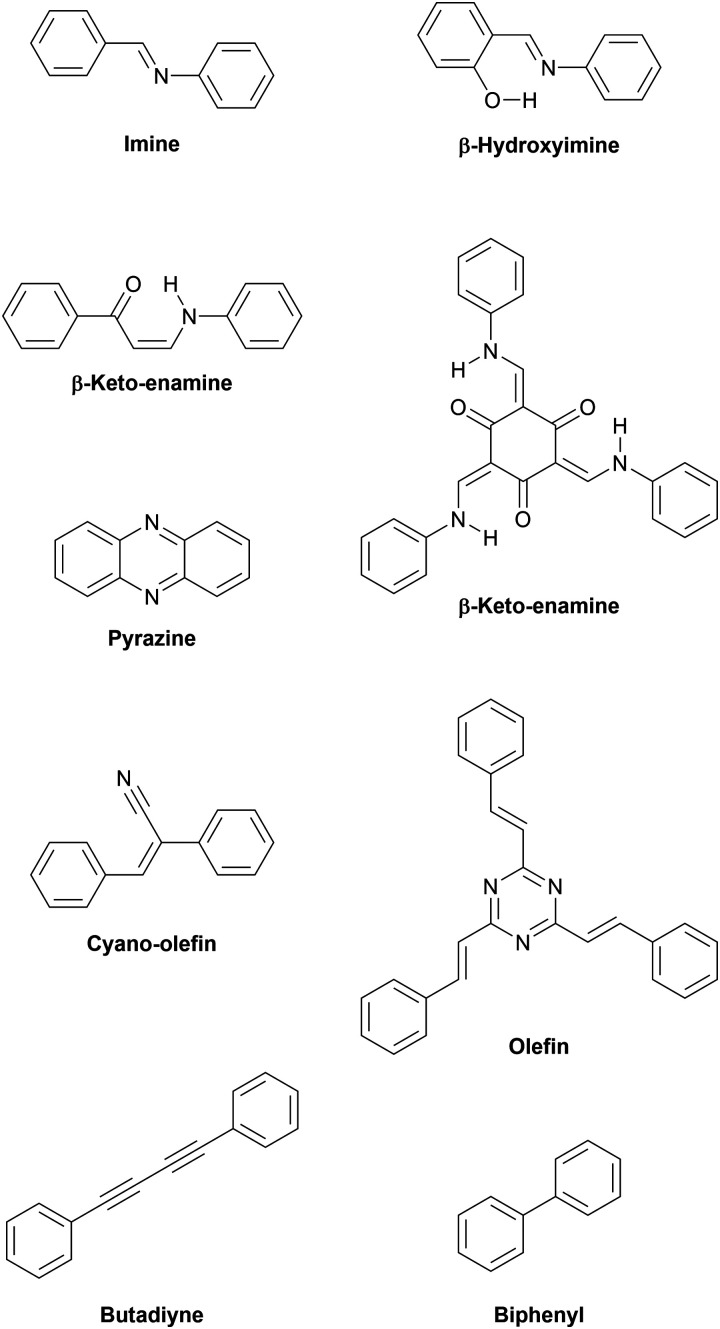
Various linkages enabling π-conjugation within layers.

##### 2.2.1.1 Imine linkages

The first COFs were connected by boronic acid-based linkages which were rather unstable under ambient conditions. Furthermore, the boronic acid moiety is not conjugated and precludes the formation of fully conjugated materials.^14^ The second generation of COFs was based on imine linkages, allowing the formation of fully conjugated sheets. At the same time the imine group provided chemical stability in presence of a wide range of solvents, under acidic pH as well as practically indefinite shelf life under ambient conditions.^73^ The addition of hydroxy groups vicinal to the iminogenic aldehyde groups imparts additional hydrolytic stability. The hydroxy group acts as hydrogen bond donor and forms a six-membered ring with the imine group, compelling the system into a more planar geometry than the unsubstituted aldehyde.^74^ This should increase the overall conjugation of the framework, however we are not aware of any study examining this effect in detail. When instead of the regularly employed 1,3,5-triformylbenzene the triply hydroxylated 1,3,5-triformyl-2,4,6-trihydroxybenzene (also known as triformylphloroglucinol) is used, the resulting materials do not just experience intramolecular hydrogen bonding but full-fledged tautomerization from enol-imine to β-keto–enamine structures.^75^

This tautomerization is a quasi-irreversible reaction, which is boon and curse at the same time. By effectively removing the imine structure from the chemical equilibrium, the products are rendered resistant even to concentrated acids and bases.^75^ Hydrogen bonding serves to guide the emerging structure to grow in plane and thus an appreciable degree of order is inherently present in the growing system. The caveat is that the crystallinity cannot improve due to the strong thermodynamic disadvantage of the reverse reaction. Some success at improving the crystallinity has been achieved by slowing the reaction through acid-modulated protonation of the amine precursors.^76^ An elegant approach demonstrated the feasibility of combining the high crystallinity of imine frameworks with the exceptional stability of β-keto–enamine frameworks. The 1,3,5-triformylbenzene vertices in a highly crystalline imine framework were postsynthetically exchanged with 1,3,5-triformyl-2,4,6-trihydroxybenzene, but the high degree of crystallinity of the imine material was retained. Following this strategy it is also possible to access keto–enamine frameworks difficult to obtain *via* direct synthesis.^77^ The exceptional chemical robustness of β-keto–enamine COFs has already been exploited for real-world applications such as reversible sensing of hydrogen gas,^78^ showcasing the suitability of these materials for further applications.

##### 2.2.1.2 Pyrazine linkage

The pyrazine unit is formed by the condensation of two vicinal ketones and two vicinal amines, effectively forming a double-imine.^79^ While the first condensation is still reversible, the second condensation locks the structure in place and imparts extra aromatic stabilization, which renders the ring closure an irreversible process. The ring closure also planarizes the system and inherently avoids the formation of defects if symmetric precursors are used exclusively. The resulting materials are fully conjugated and generally display very high bulk electrical conductivity as well as chemical stability.^79–82^ In 2019, two studies examining pyrazine-linked COFs synthesized from phthalocyanine and pyrene building blocks were reported.^80,81^ In one report, the polycrystalline, layered material exhibited similar electronic properties, such as bandgaps of 1.2 eV, bulk conductivities on the order of 10^−7^ S cm^−1^, and anisotropic hole mobilities. The out-of-plane hole mobilities were determined to be 4.8 ± 0.7 and 0.9 ± 0.2 cm^2^ V^−1^ s^−1^, whereas in-plane hole mobilities were found to be practically null.^80^ Notably, the other report showed significantly higher bulk conductivity of 2.5 × 10^−5^ S cm^−1^ for a material of similar structure, which could be raised by three orders of magnitude by iodine doping.^81^

These reports are still few but show great promise for pyrazine-based materials as active layers in electronic devices, but no charge carrier mobilities determined from OFET setups have been reported yet.

##### 2.2.1.3 Olefin linkages

In 2016, the aldol condensation of electron-deficient 1,4-phenylenediacetonitrile with a trialdehyde yielded a crystalline material connected by cyano-substituted olefin units, but in this initial report no use is made of the fully conjugated sp^2^-carbon skeleton.^83^ This concept was further explored later utilizing pyrene as tecton. The obtained layered material exhibited a bandgap of 1.9 eV as determined by cyclic voltammetry experiments. Upon doping with iodine, its electrical conductivity increased to 7.1 × 10^−2^ S m^−1^.^84^ Building on this work, a series of cyanovinylene frameworks exhibited the expected stability of a pure carbon backbone even in the presence of highly concentrated hydrochloric acid, potassium hydroxide, and aggressive organic solvents. The fully conjugated skeletons are highly emissive in bulk and as exfoliated sheets, showcasing the strong π-conjugation arising from the carbon–carbon linkage.^85^

Since the beginning of 2019, multiple reports of unsubstituted olefin-linked triazine frameworks have appeared, emphasizing the great interest in these highly conjugated linkages.^86–91^ As with their nitrile-substituted predecessors, these materials are formed *via* aldol condensations and are able to withstand even concentrated acidic as well as basic solutions for extended periods of time without apparent loss of crystallinity. The eclipsed olefin bridges in these networks are amenable to photoinduced reversible interlayer dimerization, switching the materials between a fully conjugated, two-dimensional layered structure and a non-conjugated, three-dimensional state. The effect on the electronic structure was observed by diffuse reflectance UV-Vis spectra. Two-dimensional P^2^PV and its photodimerization product, P^3^PcB, revealed optical bandgaps of 2.52 and 2.95 eV, respectively, demonstrating a significant blueshift due to breaking of the extended π-conjugation upon photodimerization.^90^ Analogously, photoinduced ring-closing reactions are employed in molecular switches in order to influence the charge injection at interfaces of multilayer devices.^92^ A switchable COF could be switched between insulating and conducting, using light and temperature as external stimuli.

##### 2.2.1.4 COFs from irreversible C–C coupling reactions

Crystalline organic polymers synthesized from irreversible such as C–C coupling reactions are not classically counted among COFs as this term has been mostly reserved for materials synthesized from reversible reactions. Lately, these lines have begun to blur as more examples of ordered structures from irreversible reactions have surfaced. Navigating irreversible C–C coupling reactions in such a way that defects are kept at a minimum is a significant challenge as no error correction can take place in these reactions.

###### 2.2.1.4.1 Graphdiynes

Graphdiynes are synthesized from arylalkynes *via* copper(ii)-mediated Glaser-type polymerization reactions, forming lattices of benzene rings connected *via* 1,3-butadiyne – often simply referred to as diyne – groups. A major obstacle associated with terminal alkynes is their inherently high reactivity, therefore alkyne monomers have a tendency to decompose under ambient conditions.^93^ During polymerization the decomposition products interfere with the structure reticulation, causing irreparable defects. To remedy this issue, the Hiyama coupling offers an elegant way to cross-couple alkynes directly from the silyl-protected state *via* an *in situ* deprotection-and-coupling cascade. A modified Hiyama coupling has recently been modified to polymerize protected alkynes to ultrathin graphdiyne films suitable for the construction of an OFET device, using graphene as well as hexagonal boron nitride as substrates.^94^ An alternative synthetic protocol uses copper foil as a physical template and as the active metal species in the network-forming polymerisation of C_3_-symmetric, organic building blocks with terminal alkynes. In theory, growth of the resulting diyne materials should self-terminate once all reactive surface functional groups on the metal support have become covered by the first complete layer(s) of the polymer. In practice, Cu(i) and Cu(ii) species coordinate strongly to alkyne functional groups of the organic building blocks and break away from the bulk metal support.^95^ Copper species become dislodged from the surface during the polymerisation reaction, and diffuse up to 20 μm away from the surface. Here, these copper species aggregate as nanoparticles (Cu(i) by XPS) and they continue to act as a quasi-homogeneous reagent that promotes further polymer growth away from the surface functional groups. The resulting polymer film is a 2D/3D van der Waals (vdW) heterostructure based on triazine (Tz, C_3_N_3_) linkers.

The most primitive member of the graphdiyne family is a carbon allotrope that consists of benzene rings in which each of the six carbon atoms is connected to the next benzene ring *via* a diyne group. The calculated bulk material properties were found to depend strongly on the stacking mode, similar to few-layer graphene. For AA stacking a metallic state is predicted, while for some AB stacking modes semiconducting behaviour is predicted. The graphdiyne monolayer is predicted to be semiconducting with a band gap of 0.46 eV and high charge carrier mobility of 10^5^ cm^2^ V^−1^ s^−1^ (Table 1).^15,96,97^ While the high mobility is important for high frequency operation and high on-state current, the sizable band gap allows for low off-state currents and therefore a high on/off ratio. An experimental study indeed found a high off-state current in a transistor setup employing a graphdiyne film synthesized on hexagonal boron nitride. The authors mention that their films might have excessive defect states, hence the relatively low field effect mobility could also be caused by defects instead of the electronic structure of ideal graphdiyne.^94^

C–C coupling reactions catalyzed or mediated by metals can yield highly cross-linked but usually amorphous polymer networks. While slowing polymerization processes down can be advantageous to avoid side reactions,^93^ it can also help improve structural order in the resulting system. We obtained an ordered triazine-based graphdiyne polymorph by Glaser coupling, whereas an analogous reaction co-catalyzed by palladium yielded an amorphous material exhibiting inferior semiconducting properties.^98^

###### 2.2.1.4.2 Cross-coupling reactions

If carried out at an interface, in principle any C–C cross-coupling reaction utilizing planar tectons can yield crystalline sheets. Restricting the reaction to a liquid–liquid interface can be accomplished by separating the components necessary to facilitate the coupling into separate phases so that coupling events can only happen where all necessary components meet, *i.e.* at the interface. For instance, the Suzuki coupling is a Pd-catalyzed cross-coupling reaction which couples arylboronic acid derivatives to aryl halides, which makes it attractive as a staging ground for the formation of conjugated materials. Its mechanism requires the presence of stoichiometric amounts of an oxygen base, typically provided by carbonate or hydroxide salts. Deliberately separating the organic precursors from the aqueous base solution provides an interface at which all required components can meet to form two-dimensional materials.^99,100^

#### 2.2.2 Large-area highly crystalline domains

The presence of grain boundaries and defects in crystalline semiconductors are detrimental to charge transport performance, hence large single-crystalline domains are aspired. We will highlight a number of different strategies to reduce defects, increase domain sizes, and strengthen interlayer interactions to obtain highly crystalline materials.

Next to synthetic approaches, one generally important aspect to consider when synthesizing porous, crystalline materials is the drying process. Two very recent studies demonstrate that the regularly used vacuum evacuation can lead to pore collapse, resulting in lower crystallinity and accessible surface area.^101,102^ It is therefore conceivable that feasible reaction conditions, especially shorter reaction times, have been obscured by inadvertent destruction of sensitive frameworks. To avoid pore collapse, a mild activation process based on a simple nitrogen flow process was proposed.^101^ Alternatively, a final washing step with low-surface tension solvent such as perfluorohexane can be carried out before vacuum drying to avoid pore collapse.^102^

##### 2.2.2.1 Modulated growth processes

In addition to stoichiometric amounts of building blocks, monodentate modulator molecules such as catechol slow the COF formation process. In one report, the addition of controlled amounts of water to condensation reactions was investigated. The authors found that small amounts of water enhanced the reversibility of the condensation reaction and increased the sizes of the resulting crystalline domains to 40 nm. Consequently, the crystalline domain area increased by a factor of 4 and the BET surface area to about 2000 m^2^ g^−1^, approaching the theoretical limit of the material in case.^103^ Similarly, addition of 10 mol% of monodentate phenylboronic acid derivatives as modulators in a microwave synthesis yielded COF-5 with an unprecedentedly high BET surface area of 2100 m^2^ g^−1^ and a remarkably high degree of crystallinity.^104^ Both of these examples are based on the boronate ester linkage, but the modulation strategy is applicable to imine frameworks as well.^105^

##### 2.2.2.2 Interlayer interactions

Studies have shown that the alternating stacking of electron-rich and electron-deficient aromatic systems can be exploited to improve stacking order in COFs. For instance, the synthesis of an imine COF using equimolar amounts of fluorinated and non-fluorinated terephthalic aldehyde gave rise to a highly crystalline framework with alternating electron-rich and electron-deficient layers.^106^ The propensity of molecules with dipole moments to align in preferred orientations can be exploited as well. Pyrene-4,5-dione-based building blocks have large dipole moments and preferably assume an antiparallel alignment. This behaviour translates into a COF, with dione units alternatingly protruding from the edges into its pores in a zig-zag fashion. Following this approach, the authors obtained a highly crystalline material with a BET surface area of 1510 m^2^ g^−1^, which is almost twice that of the non-oxidized parent pyrene COF, indicating a more highly ordered system.^107^

While intralayer hydrogen bonding has been widely employed in COF synthesis to increase chemical stability, interlayer hydrogen bonding is still relatively unexplored. In a recent example, three amide groups on the sterically encumbered central phenyl ring of a tritopic building block are forced to twist out of plane, enabling the formation of N–H–O contacts between adjacent layers. The hydrogen-bonded COFs exhibit enhanced crystallinity, improved surface area, and increased chemical stability.^108^ While the sterically induced twist in the presented system is likely to decrease the conjugation, it is conceivable that this strategy can be applied to different building blocks, preserving the conjugation while enhancing interlayer interactions as well.

Bein and coworkers introduced two concepts for the synthesis of highly crystalline imine frameworks. Both concepts rely on reducing strain within newly emerging layers and allowed the authors to obtain crystalline domains up to 500 nm in diameter.^109,110^ Due to steric constraints, molecules such as triphenylamine assume propeller-shapes with their aromatic rings – the propeller blades – tilted in the same rotational direction. If one of these tilted units forms a π-conjugated linkage with another aromatic building block, the same rotational direction is induced in the newly connected unit. If two propeller-shaped molecules are connected *via* a *C*_2_-symmetric unit, such as biphenyls, the same rotational direction is induced in both tectons. These self-repeating units serve as “molecular docking sites” for consecutive COF layers. Hence, all the tectons in newly emerging COF islands automatically assume the same rotational direction and are able to coalesce without mismatch, indepenent of their point of origin.^109^ Since the peripheral phenyl rings in tetraphenylpyrene are able to rotate independently of each other, they can assume various conformations, *e.g.* “propeller” or “armchair” conformation. In order to minimize the layer offset and maximize π-stacking of the central pyrene cores between adjacent layers, all peripheral phenyl rings throughout a stack of pyrenes are compelled to face in the same direction (“armchair”). Condensation with rigid π-conjugated units, such as terephthaldehyde, causes the peripheral phenyl rings of all connected pyrene stacks to face in the same direction. This phenomenon was dubbed “synchronized offset stacking”.^110^

#### 2.2.3 Single crystals

**Fig. 5:**
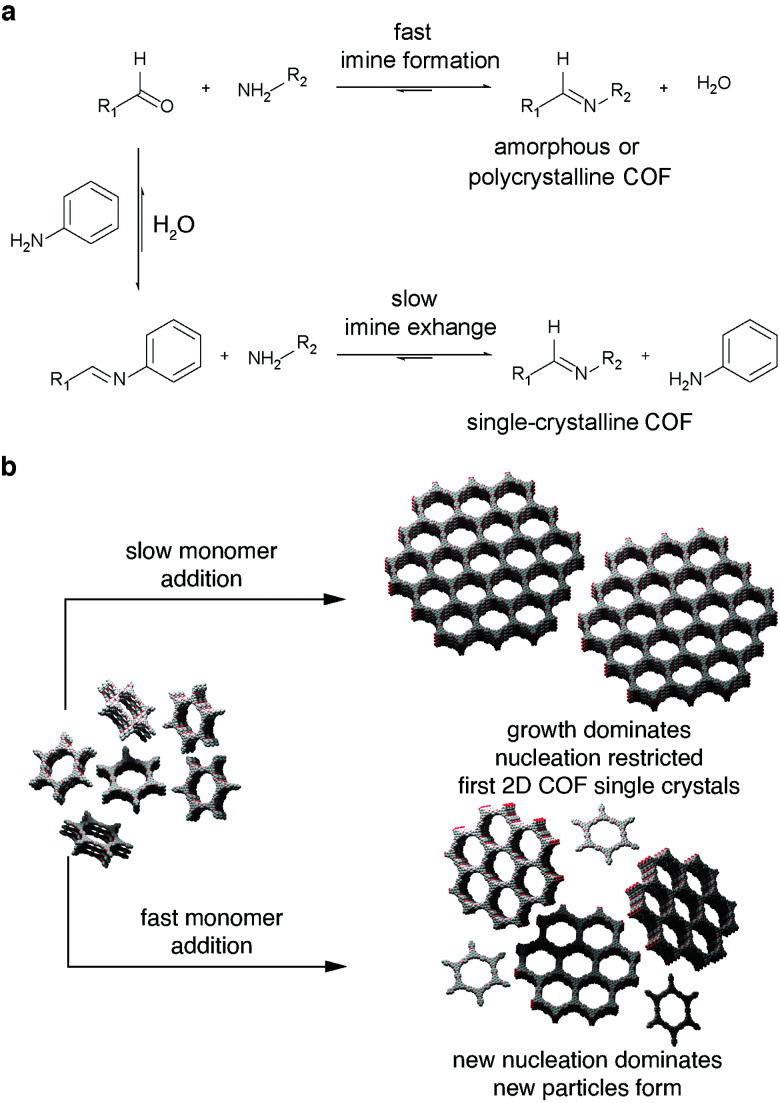
Strategies to obtain single crystal COFs; (a) slow monomer addition to pre-formed particle seeds to favor seed growth over further nucleation;^111^ (b) addition of monodentate modulator to slow growth process in order to avoid the formation of amorphous or polycrystalline phases.^105^ Adapted with permission from AAAS.

In 2018 the first two instances of single crystals amenable to X-ray diffraction were published in the same issue in Science.^105,111^ The two groups facilitated this by different means, however both strategies depended on slowing the COF particle growth. Where the group of Yaghi added a large excess of monodentate modulators (Fig. 5a),^105^ the group of Dichtel utilized slow consecutive addition of monomers to preformed particle seeds, which favored the growth of pre-existing particles rather than seeding new crystals (Fig. 5b).^111^ While one report focuses on three-dimensional imine COFs comprising tetraphenylmethane building blocks and elaborates on the various crystalline properties of the resulting single crystals,^105^ the other report features two-dimensional boronate ester-based materials and highlights their improved superior electronic properties such as (out-of-plane) charge carrier mobility due to the reduced number of defects in the structures.^111^

While both of these reports did not yield a 2D-conjugated semiconductor, they are two of the most important milestones in the development of the field and provide the necessary tools to produce conjugated, single-crystalline covalent organic frameworks. The application of the modulated imine synthesis approach is a viable route towards a 2D layered semiconductor.

#### 2.2.4 Thin films

**Fig. 6:**
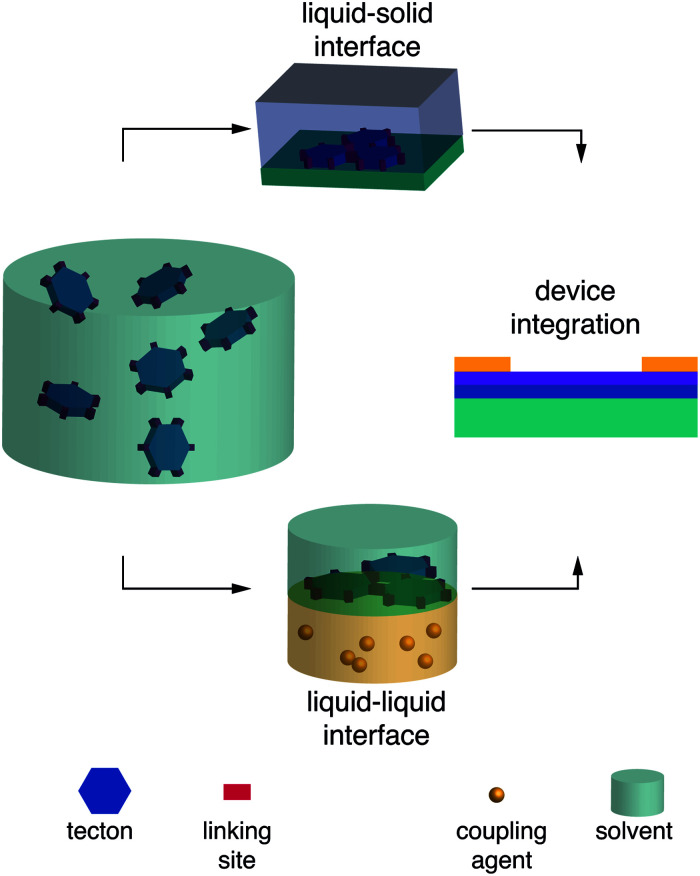
Top, liquid–solid interfacial synthesis; bottom, liquid–liquid interfacial synthesis.

COFs are classically obtained as insoluble powders, which are challenging to interface with electrodes.^112^ For successful device implementation, the generation of COFs with thin film morphology has to be achieved. Popular techniques to obtain thin films are (i) exfoliation, (ii) synthesis directly on substrate (Fig. 6, top), and (iii) synthesis at gas–liquid or liquid–liquid interfaces (Fig. 6, bottom). The thickness of surface- or interfacially grown films can be varied qualitatively as a function of reaction time and monomer concentrations. Obtaining uniformly thin films with predictable thickness, however, is challenging.^113–117^

##### 2.2.4.1 Exfoliation

Exfoliation can afford sheets for the fabrication of electronic devices. Several exfoliation methods have been reported including sonication of powders in organic solvents,^85,118–122^ mechanical exfoliation *via* grinding or ball-milling,^90,123–125^ and chemical exfoliation techniques.^126^ These methods can yield thicknesses down to the single-layer scale^120^ but typically only produce sheets up to several hundred nanometers in lateral size.

Other chemical^127–129^ and charge-induced^130,131^ exfoliation techniques have been reported but these methods are not generally applicable to other materials due to their dependence on specifically included functional groups.^127,128,130,131^ Imine COFs display copious nitrogen lone pairs amenable to protonation and charge-induced exfoliation^132,133^ but many frameworks are susceptible to amorphization at pH values sufficiently low to effect multiple protonation events.^132^ The chemical and mechanical stability of recently reported olefine COFs are far superior to imine COFs, enabling exfoliation *via* grinding as well as sonication in pure sulfuric acid to yield continuous sheets of several micrometers.^90^

These reports make it evident that various exfoliation techniques are viable to obtain few-layer materials with lateral sizes of several hundred nanometers. For especially stable materials, exfoliation under harsh conditions can produce crystalline sheets of sufficient size for device integration.^90^

##### 2.2.4.2 Solid–liquid interfaces

Surface-assisted COF syntheses have been carried out by immersion of substrates into reaction mixtures. In this manner, COF can be obtained as a film on the substrate, although the bulk of the product is still obtained as powder. Substrates for surface-assisted COF syntheses include graphene,^94,114,115,134–137^ hexagonal boron nitride,^94,116,137^ indium tin oxide,^138,139^ platinum,^140^ glass,^141^ highly oriented pyrolytic graphite,^142,143^ and molybdenum oxide.^144^ It was shown that COFs can grow on various substrates independent of lattice parameters and symmetry.^134^ In most cases the formed COF layers are oriented in parallel to the substrate surface.^94,116,134,137–139,141,144^ For conjugated materials, such as imine-linked COFs, a red-shifted UV-vis absorption edge has been observed as a result of the higher degree of conjugation in the oriented films compared to bulk powder.^116^

We have shown that copper surfaces can be used as a template and dual-role catalyst for the one-pot formation of triazine-based van der Waals heterostructures. The copper surface facilitates the cyclotrimerization of alkynes to an ordered film, which is merged with a 3D-amorphous graphdiyne phase whose formation is mediated by copper ions leached from the metal surface.^95^

The surface-assisted synthesis approach has already afforded several materials which were employed for the fabrication of OFET devices (Table 1).^94,115,116^

##### 2.2.4.3 Liquid–liquid and gas–liquid interfaces

Liquid–liquid and gas–liquid interfaces are among the smoothest surfaces known^145^ and lend themselves for monomer alignment prior to polymerization, facilitating growth in two dimensions along the interface. To perform a liquid–liquid interfacial polymerization, for example, the monomer(s) can be dissolved in one solvent and the coupling agent in a second, immiscible solvent, and the solutions carefully placed on top of each other. The interfacially grown film can then be transferred to any substrate for the fabrication of a device.

By using surfactants to support the interfacial synthesis of imine COF films, the authors obtained crystalline domains of 100–150 nm.^146^ Following the same methodology, imide COF formation was accomplished at room temperature, which is far below conventional imidization temperatures above 100 °C. The crystalline phase was estimated to constitute approximately 60% of the obtained material and contained single-crystalline domains with average sizes of 3.5 μm^2^.^117^

Mono- and multilayer porphyrin COF films were synthesized at air–water and liquid–liquid interfaces, respectively. The polycrystalline monolayer material was transferred to a SiO_2_/Si substrate for the fabrication of an OFET.^147^

After layering an organic solution containing monomers and palladium catalyst on top of a basic aqueous solution, interfacial Suzuki-type polymerizations yielded films suitable for the fabrication of OFET devices as well.^99,100^ The obtained charge carrier mobilities are among the highest values determined in a COF OFET setup.

Dichtel and coworkers were able to control the thickness of imine films obtained from Lewis acid-catalyzed liquid–liquid interfacial reactions. The resulting crystalline films ranged between 2.5 nm and 100 μm in an almost linear dependence on the initial monomer concentration.^148^

Choi and colleagues demonstrated precise control over the number of layers of imine COF films synthesized from an organic precursor solution spread on top of an aqueous phase. This was accomplished by adjusting the composition of the organic phase in order to keep the polarity on a level that precludes precipitation of the organic precursors at the interface between organic and aqueous layer. Film thicknesses between one and eight layers were obtained by varying the loading volume or concentration of the monomer solution. The lateral dimensions of the obtained films were limited by the reaction vessel, which in this case afforded wafer-size films with diameters of four inches.^149^ This method was later modified for the synthesis of pyrazine-linked COF films.^82^

## 3. Organic thin-film transistor

**Fig. 7:**
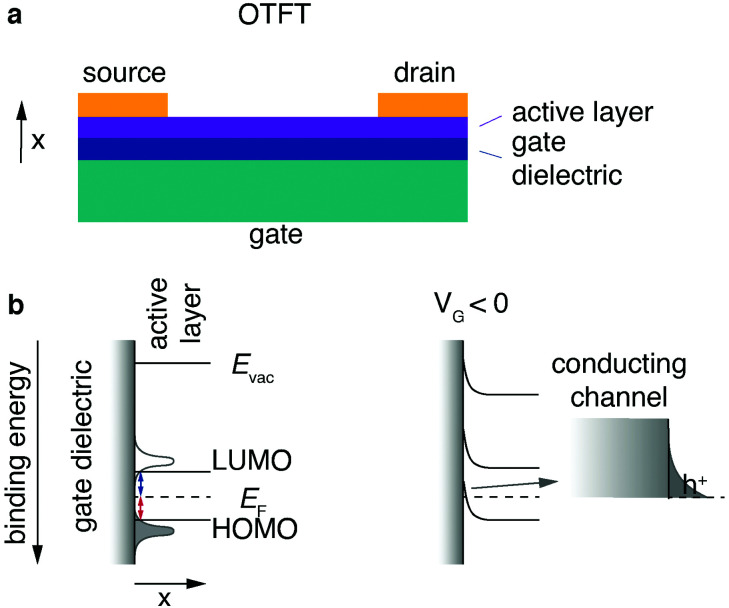
Architecture and operation of OTFT. (a) Architecture of a top-contact OTFT. (b) Energy level alignment at active layer-insulator interface in off-state and with applied gate voltage in on-state (hole conduction). The arrow marks a magnified area.

One of the most common organic field effect transistor (OFET) architectures used to characterize the electronic properties and performance of organic semiconductors is the organic thin film transistor (OTFT). The OTFT is a device comprising three terminals, namely source, drain and gate (Fig. 7a). A thin film (20–200 nm) of an organic semiconductor, called the active layer, is either spincoated or evaporated by physical vapour deposition onto what is referred to as the “gate insulator” (typically silicon dioxide). The gate insulator electrically separates the “gate” (typically doped and conductive silicon) from the active layer as well as source and drain electrodes. COF films can either be grown from solution directly on a substrate; alternatively, a thin film can be transferred onto a substrate (see Chapter 2.2.4). For a top contact device the source and drain contacts are evaporated on top of the active layer. Bottom contact devices have the source and drain contacts prefabricated directly on the gate insulator, and the active layer is deposited on top.

Typically, organic semiconducting materials show similar electron and hole mobilities. However, the surface groups of commonly used gate insulator materials – such as silicon dioxide – negatively affect the electron conduction by trapping electrons.^150,151^ Ambient water and oxygen have the same effect. For this reason, simple OTFT architectures are most often only used in p-type operation (*i.e.*, hole conduction). To enable stable n-type conduction in an organic semiconductor under ambient conditions, the onset lowest unoccupied molecular orbital energy has to be more negative than −4 eV. To reach this threshold, electron-withdrawing groups or heteroatoms have to be introduced into the structure of the semiconductor.^152,153^

Charge transport in organic small molecules and conjugated one-dimensional polymers is predominantly hopping between adjacent π-orbitals, therefore it is expedient to think of the energy levels as a Gaussian-shaped distribution of localised highest occupied molecular orbital (HOMO) and lowest unoccupied molecular orbital (LUMO) states.^154^ Even “band like” transport characteristics in small molecule single crystals cannot be equated with band transport in inorganic materials.^155^ The energy level alignment of a typical OTFT architecture without applied voltage is depicted (Fig. 7b, left). To simplify the concept, the onsets of the LUMO and HOMO are denoted as lines.

An ideal semiconducting organic material has close to no charge carriers at room temperature, and typically injection barriers are present between the organic active layer and the metal contacts (Fermi level pinning).^154^ Hole and electron injection barriers (Fig. 7, red and blue lines, respectively) are the energetic differences between the Fermi level and the onsets of HOMO and LUMO, respectively.

For an applied voltage between drain and source (*V*_DS_) the corresponding current (*I*_DS_) is therefore low; this is called the “off-state”. To switch to the “on-state”, negative gate voltage (*V*_G_) is applied between source and gate electrode resulting in an electric field arising between gate and active layer. The electric field and therefore the field effect can be maximised by decreasing the gate insulator thickness with a 2D insulator, for example with hBN or by using a high-*k* dielectric. A 2D example is using highly crystalline hBN to increase the performance of 2D transistors by providing a trap-free, thin dielectric.^156^ Its main task is, however, to inhibit current flow between gate and active layer and the result is what can be thought of as a capacitor. Positive charge carriers start to accumulate in the active material at the insulator interface (Fig. 7b, right). This local positive “charging” of the active material induces a reduction of the local Fermi level of the active layer at the insulator interface, resulting in an alignment of the transport states (HOMO) of the active layer and the electrode Fermi level. The now established energy level alignment constitutes a conductive channel for holes which can be injected from the source electrode. The described effect is synonymous to the “field effect” in organic materials and “switches” the previously non-conductive active material into a conducting channel for holes. For an applied voltage *V*_DS_ the corresponding current *I*_DS_ should be increased by orders of magnitudes compared to the off-state. Due to the necessary accumulation of charges at the interface between insulator and active layer, this mode of operation is known as “accumulation” or “enhancement”.^157^ An electron conduction mode can be established if the device architecture (no Si–OH/active layer interface) and the material (no charge traps for electrons) allow it. Transistors that function in electron as well as hole conduction mode are called ambipolar. The extraction of the field-effect mobility from the obtained *I*–*V* curves (output characteristics and transfer curves) is discussed in great detail elsewhere.^158^

Replacing the active layer of a field effect transistor with a 2D crystal has two major advantages. 3D materials like silicon stripped down to less than 3 nm thickness are strongly affected by dangling bonds. The dangling bonds constitute scattering sites which decrease the mobility of the material at these small dimensions. 2D crystals on the other hand are atomically thin without dangling bonds, therefore the problem is non-existent for this material class. The second merit of 2D crystals is their electrostatics. In short-channel field effect transistors with 3D active layers current leakage is typically induced due to poorly controllable electrostatics between electrons in the channel and the electric field applied by the gate. Whereas the channel in 3D active layers has a physical extension, in 2D crystals all carriers are confined to the atomically thin channel and the electric field can take effect more evenly. The superior gate coupling also allows to suppress current leakage if a gate voltage is applied. The culmination of these attributes could yield new low-power, highly miniaturizable device architectures like organic thin-tunneling FETs.^16^ However, processing of organic layered materials into defect-free organic 2D crystals is still in its infancy. Also, some of the materials showing high crystallinity are not fully conjugated. Employing such a material with higher interplane transport than in-plane transport can, however, still be achieved by using an alternative device architecture, the vertical field-effect transistor (VOFET).

### 3.1 Graphene-vertical organic field effect transistor (GR-VOFET)

The standard OFET architecture holds two main challenges for covalent organic solids. The first challenge is the processing of covalent organic solids into thin films for electronic device applications (see Chapter 2.2.4). The second challenge is the property of anisotropic transport. Conjugated 2D materials have different intralayer and interlayer transport properties. For instance, for TGCN it was reported that interlayer transport hopping through the stacked layers is energetically favoured compared to intralayer transport.^69^ Returning with this knowledge to the OTFT architecture, to have the maximum possible mobility the layers need to be oriented orthogonally to the source–drain axis. For standard OTFT architectures (top/bottom contact), reaching this orientation is not trivial.

**Fig. 8:**
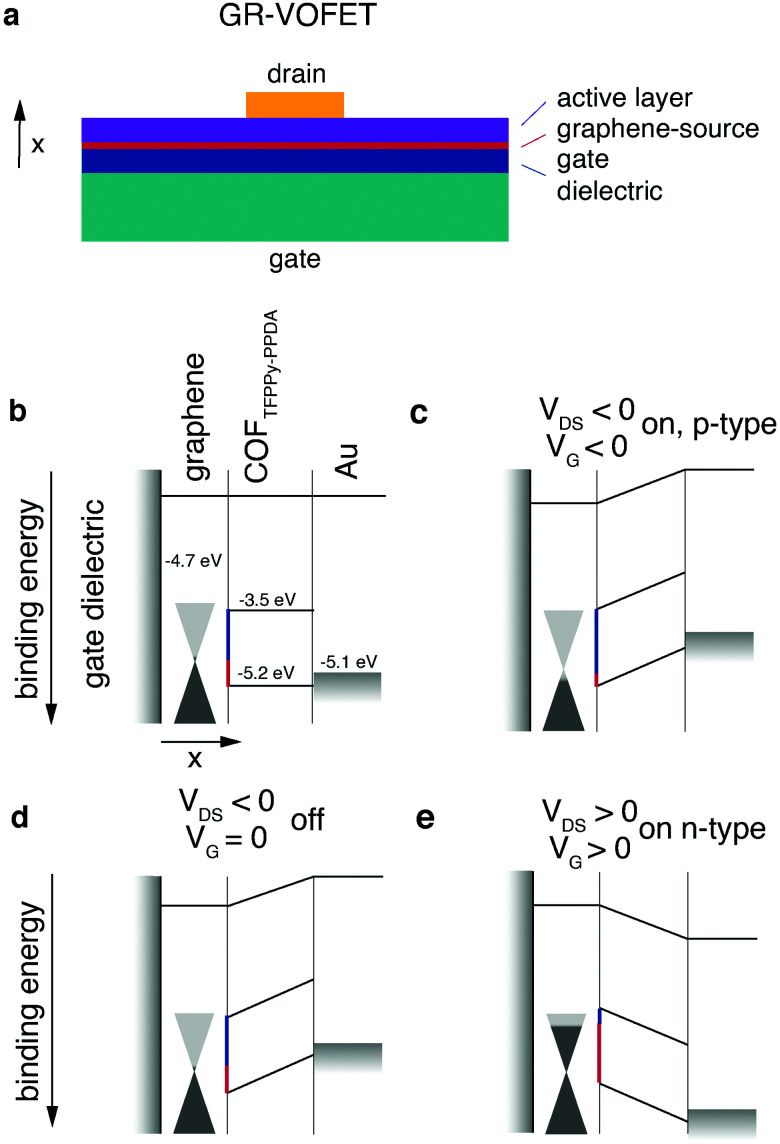
The first reported COF-GR-VOFET and its modes of operation. (a) Architecture of GR-VOFET, (b) schematic device overview, (c) hole accumulation mode, (d) without gate voltage applied, (e) electron accumulation mode. Adapted with permission from Sun *et al.*, copyright 2021 American Chemical Society.^115^

For materials exhibiting low intraplane mobility, an intriguing way to solve both of these challenges is to directly grow the crystalline material onto the graphene layer of a graphene-vertical field effect transistor (GR-VOFET, Fig. 8a). In this case the active layer is sandwiched between source and drain electrodes, the source electrode being graphene. To allow efficient hole injection into the active layer, the injection barriers between the graphene source electrode and COF layer have to be minimized (Fig. 8, injection barriers depicted in red (holes) and blue (electrons)). With no gate bias applied, the injection barrier between the source electrode and the active material is high (Fig. 8d). Upon applying a negative gate bias the local work function of graphene is increased (Fig. 8c). This is possible due to graphene's low density of states at the Fermi level as indicated by the cones touching at the tips. The increase in work function results in a decreased injection barrier to the HOMO level of the active layer. Injection of holes into the active layer is possible and the vertical source–drain channel becomes conductive, resulting in a high *I*_DS_ (Fig. 8c). Switching the gate voltage off leads to an increased hole injection barrier again (Fig. 8d). The application of a positive gate voltage results in a decrease of the work function of graphene and electron injection becomes possible (Fig. 8e). Additional benefits of this architecture are high on-current densities enabled by short channel lengths, ambipolar transport, and high on/off ratios.

The first publication presenting a COF GR-VOFET reported a current density up to 6.8 A cm^−2^ for p-type transport with a channel length of 50 nm and high on/off ratios up to 10^5^. For this device both electron and hole-accumulation mode are possible, hence it is an ambipolar transistor. The threshold voltage for the electron accumulation mode was determined to be much higher than that for hole injection. This may be due to the comparatively high electron injection barrier of 1.2 eV that has to be overcome, whereas the hole injection barrier is only 0.5 eV.^115^ While the GR-VOFET does not resolve all issues, this study showed the potential of COF materials if applied in device architectures suitable to their properties. The challenges of anisotropic charge transport, low conductivity and insolubility were resolved by a smart choice of device architecture to obtain a functional OFET device. Nevertheless, we would like to point out that the goal – a 2D organic semiconductor – can only be achieved by maximised intralayer charge transport.

## 4. Conclusion and outlook

The overarching challenge in the synthesis of layered organic materials for electronic devices is to obtain materials of the utmost purity. A similar challenge arose in the early days of semiconducting polymers where catalyst residues proved to be detrimental to device performance. This problem was largely solved by excessive postsynthetic purification of the active materials, which is not an option for covalent layered materials. Unreacted end groups cannot be removed postsynthetically and constitute structural defects deteriorating the energetic order of the materials. The goal of synthetic approaches aiming for “electronic-grade” organic semiconductors is to directly obtain the covalent scaffolds with as few structural and energetic defects as possible. For different synthetic approaches different ways of achieving “electronic-grade” purity are viable.

To obtain highly condensed, layered carbon nitride structures, ionothermal reactions yield the most crystalline and most nitrogen-rich phases to this point. The best-characterised crystalline graphitic carbon nitride material today is PTI-MX. To unlock the full potential of this material, it is now crucial to determine how to enrich and process it. Once crystalline thin films of PTI-MX can be produced reliably, the energy levels of pristine and doped species can be investigated and electrical prototype devices constructed. Due to the intercalated salt and low condensation degree the charge carrier mobility of PTI-MX is not expected to be high, but the polar point group indicates that PTI could be a piezoelectric material as observed in other carbon nitride phases.^159^ To study this property, crystalline clean surfaces of PTI have to be produced. The structure of TGCN is not researched to the same extent as the structure of PTI-MX since only nanoscale crystallites have been obtained that are accompanied by amorphous CN phases. Further complementary analysis investigating the crystal structure, elemental composition and vibrational spectra of singular crystallites are key to a better understanding of the first crystalline graphitic C_3_N_4_ material, TGCN. Theoretically predicted polymorphs such as heptazine based graphitic carbon nitride, fullerene-like as well as tube-like morphologies still have to be discovered experimentally.

Highly crystalline conjugated organic frameworks with crystalline domain sizes on the micron scale and low amounts of defects have been prepared *via* synthetic strategies that exploit universal monomer properties such as geometry, symmetry, and electron density. COF thin films can be obtained *via* exfoliation or synthesis at various interfaces, and first examples of their application in OFET devices have been presented. Despite this progress, the highest charge carrier mobility of 6.25 cm^2^ V^−1^ s^−1^ reported for an OFET with graphdiyne as the active layer falls dramatically short of the predicted mobility of 10 000 cm^2^ V^−1^ s^−1^. Generally, the highest charge carrier mobility values as determined in OFET devices are obtained for thin films with carbon–carbon linkages (Table 1). This indicates the superiority of pure carbon–carbon backbones over strongly polarized bonds such as imine linkages despite the superior materials being the products of irreversible coupling reactions and the associated defects.

Organic electronics is an interdisciplinary field requiring expertise from organic synthesis (monomers) to polymer chemistry (covalent layered materials), theory (structural and electronic order), and device fabrication. To facilitate the communication between specialists from different fields, the modes of operation of a standard OFET architecture as well as the recently introduced COF graphene-vertical OFET (GR-VOFET) architecture were elucidated. The latter illustrates how a comprehensive understanding of all aspects involved enables elegant architecture design to exploit material-specific strengths, which is high out-of-plane charge carrier transport in the case at hand.

We hope that this review gives a good perspective on metal-free, layered semiconducting materials for 2D organic devices to facilitate the entry of motivated researchers into this exciting field.

## Author contributions

DB and MGT wrote the paper and contributed equally to this work. MJB conceived the project and co-wrote the paper.

## Conflicts of interest

There are no conflicts to declare.

## Supplementary Material
